# A Case of Emphysema Post Partum

**Published:** 1826-07

**Authors:** 


12. *Emphysema Post Partum.** A woman, aged 25, was taken in labour in the evening of April 4, and the parts being very rigid, blood was taken from the arm several times, between that and the 7th, when she was delivered of a still-born child. Immediately after delivery, a violent paroxysm of coughing came on, which threatened suffocation. At noon of the same day, about ten hours after this paroxysm, her face and neck were observed to be much swelled, the swelling diffusing itself over the trunk of the body, and causing great alarm in the minds of the friends. A sensible crepitus was distinct in the parts. The cough was still troublesome, and the breathing was somewhat oppressed. Some aperient and antimonial medicines were given, and in three days the swelling, crepitus, and cough had almost ceased. From this time she had no relapse.

* Cases of emphysema from an internal cause, are rare. There are a few on record, however, where the violent straining during parturition, whether the woman screams aloud or suppresses the language of complaint, has ruptured some of the air-cells and lining membrane of the lungs, and thus permitted the escape of air into the cellular substance of the body. This kind of emphysema has always proved a mild and tractable disease, and gives way to moderate evacuations.

